# Associations Between the Cardiovascular Health Diet Index (CHDI) and Cardiometabolic Risk Factors in Brazilian Workers: A Cross‐Sectional Study

**DOI:** 10.1111/jhn.70206

**Published:** 2026-01-20

**Authors:** Estefany Mayara Sousa Araujo, Daisuke Hayashi, Daniela de Assumpção, Carla Renata Silva Andrechuk, Leila Tassia Pagamicce, Mayza Luzia dos Santos Neves, Roberta Cunha Matheus Rodrigues, Marilia Estevam Cornélio

**Affiliations:** ^1^ School of Nursing (FEnf) University of Campinas (UNICAMP) Campinas São Paulo Brazil; ^2^ Department of Nutritional Sciences The Pennsylvania State University University Park Pennsylvania USA; ^3^ Department of Gerontology School Medical Science (FCM) University of Campinas (UNICAMP) Campinas São Paulo Brazil

**Keywords:** adult, Brazilian population, cardiometabolic risk factors, diet, food, and nutrition, diet, healthy, dietary patterns

## Abstract

**Background:**

The Cardiovascular Health Diet Index (CHDI) was recently developed to assess compliance with dietary recommendations aimed at preventing cardiovascular disease and promoting cardiovascular health on a population level, based on guidance from the American Heart Association (AHA) and adapted to Brazilian dietary culture. The purpose of this study was to assess dietary quality among workers at a public university in Brazil using the CHDI and determine its association with cardiometabolic risk factors and sociodemographic data.

**Methods:**

This cross‐sectional study included 557 workers aged 20–59 years from a Brazilian public university. Dietary quality was assessed using the CHDI (score from 0 to 100 points) and the Brazilian Healthy Eating Index‐Revised (BHEI‐R) (score from 0 to 100 points), both based on data from one 24‐h dietary recall (24HR) obtained in a phone interview by a registered dietitian using the multiple‐pass method. Clinical parameters included body mass index (BMI), waist circumference, blood pressure, lipids (LDL‐c and triglycerides), fasting glucose, HbA1c, fasting insulin (used to calculate HOMA2‐IR for insulin resistance), and C‐reactive protein (CRP). Sociodemographic variables included age, sex, education level, and race/ethnicity. Associations were analyzed using modified Poisson regression models with robust variance for dichotomous outcomes and multiple linear regression models for continuous outcomes.

**Results:**

Better adherence to a heart‐healthy dietary pattern, as measured by the CHDI, was consistently associated with lower chances of having key cardiometabolic risk factors. Specifically, Poisson regression analyses showed that each additional point in the CHDI score corresponded to about a 1% lower likelihood of having excess body weight (BMI > 25 kg/m²; *β*: 0.99; *p* = 0.0102), being classified as at increased cardiovascular risk due to abdominal obesity (waist circumference; *β*: 0.99; *p* = 0.0145), or presenting elevated diastolic blood pressure (> 80 mmHg; *β*: 0.99; *p* = 0.0370). Higher scores were also associated with about a 1% lower probability of having insulin resistance (*β*: 0.99; *p* < 0.0001) and elevated C‐reactive protein levels, a marker of systemic inflammation (*β*: 0.99; *p* = 0.0207).

**Conclusion:**

The CHDI showed stronger associations with cardiometabolic risk factors than the BHEI‐R, indicating its potential as a sensitive tool for assessing diet quality in research and surveillance. Further studies in diverse populations are needed to confirm its applicability in other contexts.

## Summary

- This study is among the first to apply the Cardiovascular Health Diet Index (CHDI) to assess dietary quality in a working population, reinforcing its potential as an innovative and underutilized tool in cardiovascular health research.
- The dietary quality assessed by the CHDI showed a stronger association with cardiometabolic risk factors than the BHEI‐R, likely due to its updated approach that considers the consumption of ultra‐processed foods and distinguishes between plant and animal‐based protein sources.
- The promising results of the CHDI in assessing dietary quality suggest that it could be a valuable tool for identifying key areas requiring dietary interventions, particularly in strategies aimed at promoting cardiovascular health and preventing cardiovascular diseases.

## Graphical abstract

Graphical abstract showing a cross‐sectional study of 557 workers aged 20–59, assessing dietary quality via the Cardiovascular Health Diet Index (CDHI) and its association with cardiometabolic risk factors.

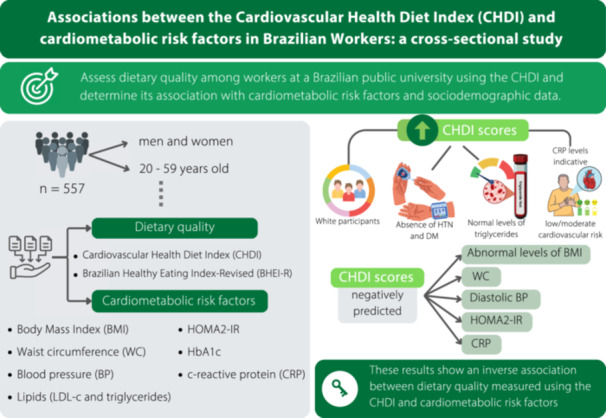

**Abbreviations**
BHEI‐R: Brazilian Healthy Eating Index‐Revised
BMI: body mass index
BP: blood pressure
CHDI: Cardiovascular Health Diet Index
CRP: C‐reactive protein
WC: waist circumference

## 1 Introduction

Cardiovascular diseases (CVDs) are the current leading cause of death worldwide, representing 38% of all premature deaths in 2019 [1]. Likewise, they are the leading cause of death in Brazil, except for the year 2021, at the height of the COVID‐19 pandemic, when deaths due to COVID‐19 infections temporarily surpassed those due to CVDs [2], leaving long‐lasting impacts on the nutritional status and well‐being of the population [3, 4]. The etiology of CVD is multifactorial, involving sociodemographic, cultural, and behavioral aspects, many of which are modifiable, such as dietary patterns, physical activity levels, and the use of tobacco‐containing products [1]. Therefore, by modifying behavioral risk factors, it is possible to improve the management and prevention of CVDs [5, 6].

Improvements in dietary quality have been associated with reductions between 14% and 23% in CVD [7, 8]. Healthy dietary patterns like the Mediterranean diet and the Dietary Approaches to Stop Hypertension (DASH) are believed to reduce the risk of CVDs by improving blood pressure and lipid profiles, as well as promoting better weight management and a healthier gut microbiota [7, 8, 9, 10]. On the other hand, dietary patterns characterized by excessive intakes of saturated fats, added sugars, salt, and ultra‐processed foods (UPF) have been linked to increases in CVDs, obesity, and type 2 diabetes [7, 8, 11]. The role of a healthy diet in cardiovascular health promotion and CVD prevention is also a key component of the American Heart Association's (AHA) Ideal Cardiovascular Health metric [12], which was introduced in 2010, emphasizing the importance of lifestyle modifications. The prevalence of Ideal Cardiovascular Health can be assessed using AHA's Life's Essential 8 (LE8) [13] metric, formerly known as Life's Simple 7 [12], and lists a healthy diet among eight components of cardiovascular health, namely not smoking, staying physically active, eating a healthy diet, proper sleep health, and healthy levels of body mass index (BMI), serum lipids, blood pressure, and fasting blood glucose [13].

Previous studies assessing the prevalence of Ideal Cardiovascular Health have suggested that adherence to a healthy diet is often the metric with the lowest prevalence rates across the studied populations [14, 15, 16]. These data indicate an important gap between dietary recommendations and people's behaviors, highlighting the need to investigate factors that may play a role in people's adherence to healthy dietary patterns, which can help inform future interventions at the individual and public health levels. However, dietary assessments are complex and require the use of tools that can reliably assess the healthfulness of people's diets [8, 17, 18]. To improve the assessment of dietary patterns, researchers have recently developed the Cardiovascular Health Diet Index (CHDI) [19], an 11‐component index based on recommendations from the AHA's recommendations for a heart‐healthy diet [20]. The CHDI was designed specifically for the Brazilian population, incorporating cultural adaptations such as the inclusion of beans and red meat, as well as a metric for UPF consumption. Unlike broader indices of overall diet quality, such as the Brazilian Healthy Eating Index‐Revised (BHEI‐R) [21], the CHDI focuses more specifically on dietary factors known to influence cardiovascular health.

The BHEI‐R [21], a 12‐component index developed in 2011 by Previdelli and colleagues based on the 2006 Brazilian Dietary Guidelines and additional evidence‐based recommendations [22, 23, 24, 25, 26], reflects the food pyramid model and evaluates some nutrients in isolation. As a result, the BHEI‐R may group foods high in sugar, sodium, refined carbohydrates, and saturated or trans fats, which contrasts with current recommendations that emphasize whole dietary patterns over individual nutrients. By incorporating measures of consumption of both cardioprotective and potentially harmful dietary components and aligning with updated recommendations for cardiovascular health, the CHDI offers a more nuanced and targeted assessment of diet quality in culturally diverse populations such as Brazil.

Despite its promising premise, the CHDI is a recently developed measure. Very few studies have employed it to assess dietary quality [19, 27, 28, 29, 30, 31], and to our knowledge, none has examined the cross‐sectional association of its scores with other indicators of cardiovascular and metabolic health. Therefore, this study aimed to assess dietary quality among workers at a Brazilian public university using the CHDI and determine its association with cardiometabolic risk factors and sociodemographic characteristics. A secondary aim was to compare CHDI scores and their associations with those obtained using the BHEI‐R.

## 2 Methods

This was an exploratory cross‐sectional study conducted as part of a larger population‐based study approved by the Institutional Review Board of a Brazilian public university located in the interior of the state of São Paulo (CAAE #28971320.5.0000.5404, Approval #3.943.389, March 30, 2020), that aimed to assess cardiovascular health indicators among workers of the same university. Data were collected between June 2022 and February 2023.

We included in the study workers of any sex aged between 20 and 59. Participants were excluded if they were on medical or maternity leave during data collection, if they declined to participate in laboratory or dietary assessments, or if they were unreachable during the data collection period after three contact attempts. Cases of “missed data collection” refer to any instance where the participant did not complete at least one major component of the study protocol (e.g., anthropometry, blood sample, or dietary recall). The sample size was calculated using the methodology for estimating a proportion [32, 33, 34]. The calculation assumed a proportion of 50%, which represents the maximum variability of the Binomial distribution in the absence of prior prevalence estimates, resulting in the most conservative (i.e., largest) sample size. A sampling error of 4% and a significance level of 5%. The total population of workers within the university based in the city of Campinas at the time of data collection was 6938 workers. To recruit a representative sample, participants invited to participate in the study were randomly selected within strata based on job category (faculty, researcher, and staff), workplace (departments within the university), and sex (male or female). The calculated sample size was 553 participants, who were randomly selected within each stratum. Participants were selected through simple random sampling within each stratum, using alphabetical lists provided by the university's human resources departments. The sampling strategy was developed with the assistance of a statistician independent from the data collection.

## 3 Measures

### 3.1 Dietary Quality

This study employed two dietary quality indices, one that measures general healthfulness and one specific to cardiovascular health. The BHEI‐R, which was developed to assess overall dietary quality within the Brazilian population [21] (an adaptation of the Healthy Eating Index‐2005 [26]) representing nine food groups, two nutrients, and the proportions of energy consumed from saturated fats, trans fats, ethanol, and added sugar. Assigning a score from 0 to 100, where higher scores represent higher dietary quality. The CHDI [19] is calculated based on 11 components: fruits, vegetables, fish and seafood, red meat, sugar‐sweetened beverages (SSB), whole grains, legumes, nuts and seeds, processed meat, dairy, and UPF. Each component is scored from 0 to 10 based on daily intake: higher scores indicate adequate consumption of healthy items or limited consumption of risk‐related items. Positive scoring is used for fruits, vegetables, fish, whole grains, legumes, daily, and nuts and seeds, while reverse scoring applies to red and processed meats, SSB, and UPF. The UPF component was scored using the NOVA score [35], which quantifies the consumption of ultra‐processed foods based on NOVA classification [36]. The total CHDI score ranges from 0 to 110, where higher scores represent better adherence to a cardioprotective diet (Supporting Information, Figure S1). Both scores were calculated using STATA 15.1 using code provided by the developers of those scores based on data from one 24‐h dietary recall (24HR) obtained in a phone interview by a registered dietitian using the multiple‐pass method (MPM) [37]. The MPM consists of five structured steps designed to reduce recall bias and improve the accuracy of reported intake. Although a single 24HR does not reflect usual intake at the individual level, it is appropriate for estimating group‐level dietary patterns in large samples [38]. To improve representativeness, recalls were collected over a 9‐month period and covered different days of the week, including weekends (Sunday to Thursday), with intentional scheduling to avoid overrepresentation of specific days of the week. This strategy, along with the randomized sampling, enhances the reliability of dietary data at the population level. To increase the accuracy of the 24HR, after each phone interview, the food portions reported by study participants were converted into units of weight or volume using a conversion table for the Brazilian population [39, 40]. Dietary intake data were entered and analyzed using Nutrition Data System for Research software version 15 (2015) developed by the Nutrition Coordinating Center (NCC), University of Minnesota, Minneapolis, MN, which provides macro and macronutrients for each food reported by participants [41].

### 3.2 Cardiometabolic Risk Factors

Anthropometrics: BMI was calculated using the formula BMI = [weight (kg)/height^2^ (m)] and classified as normal (≤ 25 kg/m²) or overweight/obesity (> 25 kg/m²). Height and weight were measured using an electronic scale with a built‐in stadiometer. Waist circumference (WC) was measured with nonelastic tape in the horizontal plane midway between the lowest ribs and the iliac crest. WCs below 94 cm (for males) and 80 cm (for females) were classified as normal.

Blood pressure (BP): BP was measured using a digital monitor based on procedures recommended by current guidelines for the Brazilian population [42]. High blood pressure was defined as systolic BP > 120 mmHg; and diastolic BP > 80 mmHg.

Biochemistry: Lipids (LDL‐c and triglycerides), fasting glycemia and fasting insulin (used to calculate HOMA2‐IR), HbA1c, and C‐reactive protein (CRP) were analyzed by a commercial laboratory. Study participants were instructed to fast for 12 h before the procedure. Values were classified as follows: HbA1c (normal: < 5.7%, high: ≥ 5.7%); triglycerides (normal: < 150 mg/dL, high: ≥ 150 mg/dL); LDL‐c (normal: < 130 mg/dL, high: ≥ 130 mg/dL); HOMA2‐IR (normal: ≤ 1.8, insulin resistance: > 1.8; CRP (low or moderate risk: ≤ 3 mg/L, high risk: > 3 mg/L).

Medical history: Participants reported whether they had been previously diagnosed with hypertension, dyslipidemia, and/or diabetes mellitus.

### 3.3 Sociodemographic Data

Study participants provided the following sociodemographic data: sex (male or female), age (years), race (White or other), education (years of study), and marital status (partnered or single).

### 3.4 Procedures

Data were collected between June 2022 and February 2023 by four trained researchers (three nurses and one registered dietitian). Data collection was done in three parts: one (virtual), where prospective participants were emailed an invitation to participate in the study accompanied by a link to a virtual consent form, followed by a sociodemographic and medical history questionnaire, as well as an online schedule where they could choose a date and time for the second part of data collection. Two (in person), where participants showed up at the university's community health services, where they had their BP and anthropometrics measured, and their blood samples drawn. Three (phone), where a researcher interviewed each participant to collect a 24‐h food recall.

### 3.5 Data Analyses

Descriptive data analysis was carried out using frequencies, percentages, measures of central tendency (mean and median), and dispersion (standard deviation and interquartile range). The Shapiro–Wilk test was used to assess data distribution. Comparisons of a qualitative variable with two categories in relation to quantitative variables were performed using the unpaired Student's *t*‐test or the Mann–Whitney test, according to the data distribution. Multiple linear regression models using generalized linear models were carried out to identify the factors related to the quantitative outcomes [43]. Modified multiple Poisson regression models with robust variance were adjusted for the dichotomous outcomes [44], the use of these models is an alternative to traditional logistic regression. The statistical software SAS 9.4 and SPSS 25 were used for the analyses, and a 5% significance level was considered.

## 4 Results

Of the 5797 workers invited by email, 707 agreed to participate in the study, and 557 completed all three phases of data collection and were therefore included in the analyses. The sociodemographic and clinical characteristics of the study sample are presented in Table 1. Overall, 75.7% (*n* = 421) of participants self‐identified as white, 71.5% (*n* = 394) reported being partnered, and the mean duration of formal education was 18.1 years (SD = 4.21).

**TABLE 1:** Sociodemographic and clinical characteristics of workers from a Brazilian public university (*n* = 557). Campinas, June 2022 to February 2023.

Sociodemographic characteristics	Total (*n* = 557)	
	Mean	SD
Age (years)	44.9	8.72
Education (years) (*n* = 545)	18.1	4.21
	*n*	%
Sex		
Male	243	43.6
Female	314	56.4
Race		
White	421	75.7
Other	135	24.3
Marital status		
Partnered	394	71.5
Single	154	28.5
Clinical characteristics	Mean	SD
BMI (kg/m^2^)	27.32	5.48
Waist circumference (cm)	92.01	14.31
Systolic BP (mmHg)	120.03	13.59
Diastolic BP (mmHg)	74.21	9.42
	*N*	%
Hypertension	122	22.3
Dyslipidemia	170	31.3
Diabetes mellitus	34	6.3
BMI		
≤ 25 kg/m²	224	40.2
> 25 kg/m²	333	59.8
Waist circumference		
Normal	217	38.9
Higher risk	340	61.1
LDL cholesterol		
< 130 mg/dL	396	71.1
≥ 130 mg/dL	161	28.9
HbA1c		
< 5.7%	405	72.7
≥ 5.7%	152	27.3
Triglycerides		
< 150 mg/dL	456	81.9
≥ 150 mg/dL	101	18.1
HOMA2‐IR		
≤ 1.8	207	37.2
> 1.8	350	62.8
CRP		
Low/average	408	73.3
High	149	26.7

Abbreviations: BMI, body mass index; BP, blood pressure; CRP, C‐reactive protein; HbA1c, glycated hemoglobin; HOMA2‐IR, homeostatic model assessment for insulin resistance; SD, standard deviation.

A previous diagnosis of hypertension, dyslipidemia, and diabetes mellitus was reported by 22.3% (*n* = 122), 31.3% (*n* = 170), and 6.3% (*n* = 34) of participants, respectively. The average BMI was 27.32 kg/m^2^ (SD = 5.48) and 61.1% (*n* = 340) had a WC above normal. Elevated LDL‐c, HbA1c, and triglycerides were observed in 28.9% (*n* = 161), 27.3% (*n* = 152), and 18.1% (*n* = 101) of participants, respectively. HOMA2‐IR was indicative of insulin resistance in 62.8% (*n* = 350) (Table 1).

Regarding diet quality, the mean CHDI score was 49.5 (SD = 16.7) out of 110, while the mean BHEI‐R score was 61.6 (SD = 11.9) out of 100 (Table 2). The component scores are presented in Table 2, while Supporting Information Figure S2 displays radar plots as an alternative visual representation of these data. In the CHDI, eight components had mean scores ≤ 5 out of 10, including fish and seafood (1.6; SD = 3.6), nuts and seeds (2.1; SD = 3.8), whole grains (3.2; SD = 3.9), fruits (3.9; SD = 3.7), legumes (4.1; SD = 3.8), and dairy (4.4; SD = 3.8), all reflecting low consumption, as well as SSB (4.5; SD = 4.7) and red meat (4.6; SD = 4.9), indicating high consumption. In the BHEI‐R, components with mean scores below 50% of their maximum included whole grains (1.4/5) and milk/dairy (5.0/10), reflecting low consumption, and sodium (2.9/10) and calories from solid fats, alcohol, and added sugars (SoFAAS) (10.1/20), both indicating high intake (Table 2) (Supporting Information, Figure S2).

**TABLE 2:** Cardiovascular Health Diet Index (CHDI) and Brazilian Healthy Eating Index‐Revised (BHEI‐R) scores of workers from a Brazilian public university (*n* = 557). Campinas, June 2022 to February 2023.

Component	Total (*n*)	Mean	SD	Median	IQR
CHDI					
Overall score (0–110)	557	49.5	16.7	49.1	23.2
Fruits (0 a 10)		3.9	3.7	2.7	7.4
Vegetables (0 a 10)		5.9	3.3	5.6	6.5
Fish and seafood (0 a 10)		1.6	3.6	0.0	0.0
Red meat (0 a 10)		4.6	4.9	0.0	10
SSB (0 a 10)		4.5	4.7	2.1	10
Whole grains (0 a 10)		3.2	3.9	0.8	6.6
Legumes (0 a 10)		4.1	3.8	4.0	7.0
Nuts and seeds (0 a 10)		2.1	3.8	0.0	1.4
Processed meats (0 a 10)		5.7	4.8	10	10
Dairy (0–10)		4.4	3.8	3.2	7.9
UPF (0 a 10)		9.1	1.2	10	2.1
BHEI‐R					
Overall score (0–100)	557	61.6	11.9	62.1	17.8
Total fruit (0 a 5)		3.1	2.1	4.1	4.3
Whole fruits (0 a 5)		3.1	2.2	4.8	5.0
Total vegetables (0 a 5)		4.5	1.1	5.0	0.0
Dark green and orange vegetables (0 a 5)		4.1	1.7	5.0	0.0
Total grains (0 a 5)		4.2	1.1	4.9	1.2
Whole grains (0 a 5)		1.4	1.7	0.6	2.5
Milk and dairy (0 a 10)		5.0	3.5	4.7	6.2
Meat, eggs, and legumes (0 a 10)		8.7	2.3	10	1.4
Oils (0 a 10)		9.1	2.6	10	0.0
Sodium (0 a 10)		2.9	2.5	2.7	4.1
Saturated fats (0 a 10)		5.1	3.6	5.6	6.9
SoFAAS (0 a 20)		10.1	5.9	10.8	9.1

Abbreviations: BHEI‐R, Brazilian Healthy Eating Index‐Revised; CHDI, Cardiovascular Health Diet Index; IQR, interquartile range; SD, standard deviation; SoFAAS, calories from solid fats, alcohol, and added sugar; SSB, sugar‐sweetened beverages; UPF, ultra‐processed foods.

Concerning the relationship between CHDI and BHEI‐R scores and sociodemographic factors, comparison analyses showed that median CHDI and BHEI‐R scores were significantly higher among White participants compared to non‐White participants (CHDI: 50.3 vs. 44.6, *p* = 0.0052; BHEI‐R: 62.9 vs. 59.7, *p* = 0.0183) (Supporting Information, Table S1).

Multiple linear regression analyses confirmed these associations: being White was associated with a 3.80‐point higher CHDI score compared to being non‐White (*p* = 0.0264), and each additional year of education was associated with a 0.69‐point higher CHDI score (*p* = 0.0001). No sociodemographic variables were significantly associated with BHEI‐R scores in the adjusted models (Table 3).

**TABLE 3:** Multiple linear regression models between sociodemographic/clinical variables and either Cardiovascular Health Diet Index (CHDI) or Brazilian Healthy Eating Index‐Revised (BHEI‐R) scores. Campinas, June 2022 to February 2023.

Dependent variable	Independent variable	*β*	CI 95%	*p* *
CHDI (*n* = 504)	Education	0.69	(0.34 a 1.04)	**0.0001**
	Race (ref. = White)	−3.80	(−7.16 a −0.45)	**0.0264**
	Sex (ref. = M)	0.15	(−2.81 a 3.11)	0.9200
	Age	−0.02	(−0.19 a 0.15)	0.8097
	Hypertension (ref. = no)	−3.34	(−6.96 a 0.27)	0.0699
	Diabetes (ref. = No)	−4.70	(−10.81 a −1.4)	0.1313
BHEI‐R (*n* = 504)	Education	0.09	(−0.17 a 0.34)	0.5132
	Race (ref. = White)	−2.23	(−4.7 a 0.24)	0.0765
	Sex (ref. = M)	1.31	(−0.86 a 3.49)	0.2369
	Age	0.09	(−0.04 a 0.21)	0.1815
	Hypertension (ref. = no)	−3.21	(−5.87 a −0.55)	**0.0180**
	Diabetes (ref. = No)	−3.13	(−7.63 a −0.55)	0.1723

Abbreviations: *β*, linear regression coefficient; BHEI‐R, Brazilian Healthy Eating Index‐Revised; CHDI, Cardiovascular Health Diet Index; CI, confidence interval.

* Bold text means *p* < 0.05.

Regarding the association between CHDI and BHEI‐R scores and cardiometabolic factors, comparison analyses showed that median CHDI scores were significantly lower among participants without previously diagnosed hypertension or diabetes compared to participants with those disease (without hypertension 50.8 vs. with hypertension 44.1, *p* = 0.0052) (without DM 49.7 vs. with DM 43.3, *p* = 0.0405), as well as among those with normal parameters of triglycerides ( < 130 mg/dL 50.0 vs. ≥ 130 mg/dL 44.6, *p* = 0.0108), WC (normal 52.1 vs. risk 47.0, *p* = 0.0352), or elevated CRP levels (low/moderate 50.8 vs. elevated 45.9, *p* = 0.0276). For the median BHEI‐R scores, analyses showed that the scores were only significantly lower among participants with hypertension (60.2 vs. 62.3, *p* = 0.0171) (Supporting Information, Table S1). This was confirmed in the linear regression analysis, where the presence of hypertension was associated with a mean reduction of 3.21 points in the BHEI‐R score compared to individuals without hypertension (*p* = 0.018) (Table 3).

Modified Poisson regression models with robust variance (Table 4) showed that each 1‐point increase in CHDI score was associated with approximately a 1% lower probability of having excess body weight (*p* = 0.0102), being classified as at increased cardiovascular risk due to abdominal obesity (*p* = 0.0145), or presenting elevated diastolic blood pressure (*p* = 0.0370). Higher CHDI scores were also linked to about a 1% lower probability of having insulin resistance (*p* < 0.0001) and elevated CRP levels, a marker of systemic inflammation (*p* = 0.0207). For the BHEI‐R, associations with these outcomes were weaker and less consistent; an exception was a small inverse association with elevated LDL‐cholesterol (*p* = 0.0368) (Table 4).

**TABLE 4:** Multiple Poisson regression models between categorical clinical variables and either Cardiovascular Health Diet Index (CHDI) or Brazilian Healthy Eating Index‐Revised (BHEI‐R) scores. Campinas, June 2022 to February 2023.

Dependent variable	Independent variable	PR	CI 95%	*p* *
BMI (> 25 kg/m²)	CHDI	0.99	(0.99–0.99)	**0.0102**
	BHEI‐R	1.00	(0.99–1.00)	0.5092
WC (above normal)	CHDI	0.99	(0.99–0.99)	**0.0145**
	BHEI‐R	1.00	(1.00–1.01)	0.3859
HOMA2‐IR (> 1.8)	CHDI	0.99	(0.99–0.99)	**< 0.0001**
	BHEI‐R	1.00	(1.00–1.01)	0.6510
LDL (≥ 130 mg/dL)	CHDI	1.01	(1.00–1.02)	0.0697
	BHEI‐R	0.99	(0.98–0.99)	**0.0328**
CRP (high)	CHDI	0.99	(0.98–0.99)	**0.0207**
	BHEI‐R	1.00	(0.99–1.10)	0.9220
Systolic BP (> 120 mmHg)	CHDI	1.00	(0.99–1.00)	0.5495
	BHEI‐R	1.00	(0.99–1.00)	0.2439
Diastolic BP (> 80 mmHg)	CHDI	0.99	(0.98–0.99)	**0.0370**
	BHEI‐R	0.99	(0.98–1.01)	0.3843

Abbreviations: BHEI‐R, Brazilian Healthy Eating Index‐Revised; BP, blood pressure; BMI, body mass index; CHDI, Cardiovascular Health Diet Index; CRP, C‐reactive protein; HOMA2‐IR, homeostatic model assessment for insulin resistance; IC, confidence interval; PR, prevalence ratio; WC, waist circumference.

* Bold text means *p* < 0.05.

## 5 Discussion

This study aimed to assess diet quality among workers at a public university in Brazil and examine the relationship between diet quality assessed with either a recently developed measure specific to cardiovascular health (CHDI) or a more traditional measure of overall diet quality (BHEI‐R) with cardiometabolic risk factors and sociodemographic characteristics. Our results indicate an overall poor dietary quality among the studied population when using either measure, as both of them showed lower scores than previous studies conducted with the Brazilian population [19, 30, 45]. These patterns, however, are aligned with global trends, as those highlighted in the systematic analysis for the 2021 Global Burden of Disease (GDB) [46], in which 37.6% of the population worldwide was estimated to have low consumption of fresh fruits and vegetables, whole grains, dairy, calcium‐rich foods, polyunsaturated fats, and high consumption of red or processed meats, SSB, trans fats, and sodium—variables that impact both BHEI‐R and CHDI scores.

The statistically significant associations for each measure of dietary quality, however, were different. Regarding sociodemographic and clinical characteristics, CHDI scores were positively associated with being White and having more years of education, but not with having a previous diagnosis of diabetes or hypertension, whereas median BHEI‐R scores were significantly higher among White participants compared to non‐White participants and were negatively associated with having a previous diagnosis of hypertension. Additionally, CHDI scores were significantly and inversely associated with several cardiometabolic risk factors examined in this study, namely, BMI, WC, HOMA2‐IR, CRP, and diastolic BP. The BHEI‐R, on the other hand, was only significantly and inversely associated with LDL‐cholesterol levels. Together, these data seem to suggest that the CHDI might be more sensitive when aiming to identify dietary patterns that are associated with worse levels of cardiometabolic risk factors, which might be due to its emphasis on assessing the consumption of foods known to impact cardiovascular health. For example, the CHDI has an updated approach to analyzing the consumption of ultra‐processed foods, and it discriminates between plant‐based protein‐rich foods, such as legumes, and animal‐based sources of protein, such as meats. Unhealthy diets, characterized by high energy density, excessive intake of red meat, processed and ultra‐processed foods, saturated and trans fats, sodium, and added sugars, and low in dietary fiber, are known to negatively affect cardiovascular biomarkers and anthropometric indicators, contributing to increased lifetime risk of developing CVDs [47, 48, 49, 50]. In this sense, employing the newer metric (CHDI) to assess dietary quality might be particularly beneficial in identifying the need for interventions to promote change in eating behaviors when aiming to modify cardiometabolic risk factors.

In our analyses, White participants and those with more years of education had higher CHDI (but not BHEI‐R) scores, which likely reflects the impact of social determinants of health, since race and access to education may impact and shape food access, affordability, health literacy, and culturally mediated food practices, all of which influence dietary choices [51, 52]. Evidence from large Brazilian and international cohorts supports these links. For example, Cacau et al. [28] examined dietary patterns and their sociodemographic correlates using three diet quality indices (the Planetary Health Diet Index—PHDI, the Healthy Eating Index‐2015—HEI‐2015, and the CHDI), finding that higher mean scores across all three diet‐quality indices were observed among women, older adults, individuals with greater per‐capita income, and those reporting moderate‐to‐vigorous physical activity. However, the indices showed no consistent agreement regarding race/skin color. Interestingly, our findings are only partially consistent with those from the 2015 Health Survey of São Paulo study [45], conducted in Brazil, which included 1888 participants. In the aforementioned study, being White and higher income were positively associated with dietary quality, but higher levels of education were inversely associated with dietary quality. On the other hand, a large study conducted by Xu et al. [53] in the US that included 10,808 middle‐aged adults found positive associations between being Female, older, and having higher levels of education.

The heterogeneity in findings between our study and the two studies mentioned above can be interpreted in different ways. First, it is important to notice that the methods to assess dietary quality were different in the three studies. Our study assessed food intake with one 24HR and dietary quality with the CHDI and the BHEI‐R. In contrast, the 2015 Health Survey of São Paulo [45] employed two non‐consecutive 24HR and assessed dietary quality using the BHEI‐R, while Xu et al.'s study [53] employed a food frequency questionnaire (FFQ) and assessed diet quality using the Healthy Eating Index‐2015 (HEI‐2015) and the Alternative Healthy Eating Index‐2010 (AHEI‐2010), and Cacau et al. [28] likewise applied an FFQ and examined dietary patterns using the PHDI, the HEI‐2015, and the CHDI. These methodological differences limit the extent to which the findings can be compared across studies.

In addition, contextual and conceptual aspects may also help explain the heterogeneity in findings. Social determinants of health, such as race and education, may influence diet quality differently depending on the setting and population studied [51]. Furthermore, although both the CHDI and the BHEI‐R are measures of diet quality, they differ in their structure and focus. The CHDI was specifically designed to assess the cardioprotective potential of dietary patterns, with separate components for processed meats, ultra‐processed foods, nuts and seeds, and fish and seafood, allowing for a more detailed evaluation of foods directly related to cardiovascular risk. In contrast, the BHEI‐R reflects overall diet quality but groups foods differently, for example, by combining meats, eggs, and legumes into a single component, and including total grains as a broad category that mixes refined grains, roots, and tubers. These differences in component definition and scoring may partly explain why associations with sociodemographic and cardiometabolic factors were more consistent for the CHDI in our analyses.

All in all, our findings point to different patterns of associations between dietary quality and clinical, social, and demographic variables, depending on the metric used to assess dietary quality. Overall, the CHDI was significantly associated with several of those variables (i.e., years of education, race, BMI, WC, HOMA2‐R, CRP, and diastolic BP), whereas the BHEI‐R was only significantly associated with having a previous diagnosis of hypertension and having high serum levels of LDL‐cholesterol. These results might suggest that the CHDI is more reflective of cardiometabolic risk factors and the nuanced differences in dietary patterns that arise from social and demographic determinants of health, thus supporting the relevance of this recently developed metric as part of a comprehensive assessment of behavioral determinants of cardiovascular health. This could have important implications for public health in regards to employing novel metrics to assess diet quality, and future studies should compare associations between CHDI scores and clinical and sociodemographic variables with associations between such variables and more traditional metrics of dietary quality among diverse populations and demographic groups.

It is important to emphasize that a healthy diet is not the only factor influencing cardiovascular health or its key determinants, such as BMI, WC, blood pressure, blood glucose, and lipid profile, which were evaluated in this study. Other elements included in the AHA's Life's Essential 8, such as nicotine exposure, physical activity, and sleep health, are also crucial for achieving cardiovascular well‐being [13, 15]. However, evidence shows that diet tends to have one of the lowest adherence rates among these components [14, 54, 55], highlighting the relevance of this study and the need for greater investment to facilitate population access to high‐quality and sustainable diets [30, 56, 57]. This is particularly important considering that foods and dietary elements that promote a healthier and more sustainable diet are often more expensive, which can exacerbate sociodemographic, ethnic, and racial disparities in diet quality and health outcomes.

Our study design had limitations that limit the generalizability of its findings. For instance, the cross‐sectional design does not allow for inferences regarding causality between CHDI scores and the clinical parameters indicative of cardiometabolic health assessed in this study. Additionally, the population included in this study (workers from a public university in the state of São Paulo, Brazil) is composed of individuals with relatively high levels of education and socioeconomic status compared to the average population in Brazil. Furthermore, the use of a single 24HR does not reflect long‐term dietary patterns on an individual level, and the lack of assessment of the addition of table salt to homemade culinary preparations limits the accuracy of our data on sodium intake. There are, however, important strengths in our study design, including the collection of 24HR data across participants on different days of the week, including weekends, and over 9 months, which allows our data to reflect sample‐level dietary patterns. Also, our representative and randomized sampling strategy allows for an accurate representation of the population of workers included in this study.

## 6 Conclusions

Our findings indicate that dietary quality assessed with the CHDI, a novel measure of dietary quality based on recommendations from the AHA for the prevention of CVDs, is more closely related to clinical parameters of cardiometabolic risk factors than diet quality assessed using the BHEI‐R (the Brazilian version of the Healthy Eating Index) in the population included in this study. Additionally, the CHDI also demonstrated greater sensitivity in detecting differences in diet quality across racial and ethnic groups and among individuals with varying levels of education, highlighting its potential for capturing the influence of social and demographic determinants of dietary patterns. These findings position the CHDI as a promising tool for assessing dietary quality and identifying the needs for interventions when focusing on the promotion of cardiovascular health and the prevention of CVDs, particularly in population‐level research and surveillance. While it also shows potential for application in clinical practice and primary care, further studies in different populations and settings are needed to better establish its applicability, reliability, and practical value.

## Author Contributions

Conceptualization: Roberta Cunha Matheus Rodrigues, Marilia Estevam Cornélio, Carla Renata Silva Andrechuk, Daisuke Hayashi, Estefany Mayara Sousa Araujo, Leila Tassia Pagamicce, and Mayza Luzia dos Santos Neves. Methodology: Roberta Cunha Matheus Rodrigues, Marilia Estevam Cornélio, Carla Renata Silva Andrechuk, Daisuke Hayashi, Estefany Mayara Sousa Araujo, Daniela de Assumpção, Leila Tassia Pagamicce, and Mayza Luzia dos Santos Neves. Investigation: Estefany Mayara Sousa Araujo, Carla Renata Silva Andrechuk, Leila Tassia Pagamicce, and Mayza Luzia dos Santos Neves. Data curation: Estefany Mayara Sousa Araujo and Carla Renata Silva Andrechuk. Formal analysis: Daniela de Assumpção and Estefany Mayara Sousa Araujo. Writing – original draft: Estefany Mayara Sousa Araujo, Daisuke Hayashi, Marilia Estevam Cornélio, and Daniela de Assumpção. Writing – review and editing: all authors. Project administration: Carla Renata Silva Andrechuk. Supervision, visualization and funding acquisition: Roberta Cunha Matheus Rodrigues and Marilia Estevam Cornélio. All authors have read and agreed to the published version of the manuscript.

## Conflicts of Interest

The authors declare no conflicts of interest.

## Peer Review

The peer review history for this article is available at https://www.webofscience.com/api/gateway/wos/peer-review/10.1111/jhn.70206.

## Supporting information


**Figure S1:** Components and scoring criteria of the Cardiovascular Health Diet Index (CHDI) and the Brazilian Healthy Eating Index‐Revised (BHEI‐R). **Figure S2:** Radar plot of dietary index component scores. **Table S1:** Comparison analyses between sociodemographic/clinical variables and either Cardiovascular Health Diet Index (CHDI) or Brazilian Healthy Eating Index‐Revised (BHEI‐R) scores. Campinas, June 2022 to February 2023.

## Data Availability

The data that support the findings of this study are available from the corresponding author upon reasonable request.

## References

[1] World Health Organization. Cardiovascular Diseases (CVDs). Fact Sheet. WHO Fact Sheet 2021, https://www.who.int/news-room/fact-sheets/detail/cardiovascular-diseases-(cvds), (Accessed September 10, 2023).

[2] G. M. M. Oliveira , L. C. C. Brant , C. A. Polanczyk , et al., “Estatística Cardiovascular – Brasil 2023,” Arquivos Brasileiros de Cardiologia 121 (2024): 1–131, https://doi.org/10.36660/abc.20240079. 10.36660/abc.20240079 PMC11185831 38896747

[3] H. Abdollahi , F. Salehinia , M. Badeli , et al., “The Biochemical Parameters and Vitamin D Levels in ICU Patients With COVID‐19: A Cross‐Sectional Study,” Endocrine, Metabolic & Immune Disorders ‐ Drug Targets 21 (2021): 2191–2202, 10.2174/1871530321666210316103403. 33726658

[4] H. Abdollahi , H. Tavakoli , Y. Mojtahedi , et al., “Evaluation of Depression, Anxiety and Stress Scores in Patients With COVID‐19: A Cross‐Sectional Study,” Archives of Anesthesiology and Critical Care 10 (2024): 565–570, 10.18502/AACC.V10IS2.17213.

[5] World Health Organization. Noncommunicable Diseases Progress Monitor 2020 2020:224, 10.5005/jp/books/11410_18.

[6] S. Yusuf , S. Hawken , S. Ôunpuu , et al., “Effect of Potentially Modifiable Risk Factors Associated With Myocardial Infarction in 52 Countries (the INTERHEART Study): Case‐Control Study,” Lancet 364 (2004): 937–952, 10.1016/S0140-6736(04)17018-9. 15364185

[7] K. S. Petersen and P. M. Kris‐Etherton , “Diet Quality Assessment and the Relationship Between Diet Quality and Cardiovascular Disease Risk,” Nutrients 13 (2021): 4305, 10.3390/NU13124305. 34959857 PMC8706326

[8] A. Diab , L. N. Dastmalchi , M. Gulati , and E. D. Michos , “A Heart‐Healthy Diet for Cardiovascular Disease Prevention: Where Are We Now?,” Vascular Health and Risk Management 19 (2023): 237–253, 10.2147/VHRM.S379874. 37113563 PMC10128075

[9] E. Ramón‐Arbués , B. Martínez‐Abadía , J. M. Granada‐López , E. Echániz‐Serrano , I. Huércanos‐Esparza , and I. Antón‐Solanas , “Association Between Adherence to the Mediterranean Diet and the Prevalence of Cardiovascular Risk Factors,” Revista Latino‐Americana de Enfermagem 28 (2020): 1–8, 10.1590/1518-8345.3904.3295. PMC7282722 32520245

[10] M. Rahimlou , N. Grau , N. Banaie ‐Jahromi , et al., “Association of Adherence to the Dietary Approach to Stop Hypertension and Mediterranean Diets With Blood Pressure in a Non‐Hypertensive Population: Results From Isfahan Salt Study (ISS),” Nutrition, Metabolism, and Cardiovascular Diseases 32 (2022): 109–116, 10.1016/J.NUMECD.2021.09.029. 34893410

[11] F. Juul , A. L. Deierlein , G. Vaidean , P. A. Quatromoni , and N. Parekh , “Ultra‐Processed Foods and Cardiometabolic Health Outcomes: From Evidence to Practice,” Current Atherosclerosis Reports 24 (2022): 849–860, 10.1007/s11883-022-01061-3. 36070170

[12] D. M. Lloyd‐Jones , Y. Hong , D. Labarthe , et al., “Defining and Setting National Goals for Cardiovascular Health Promotion and Disease Reduction: The American Heart Association's Strategic Impact Goal Through 2020 and Beyond,” Circulation 121 (2010): 586–613, 10.1161/CIRCULATIONAHA.109.192703. 20089546

[13] D. M. Lloyd‐Jones , N. B. Allen , C. A. M. Anderson , et al., “Life's Essential 8: Updating and Enhancing the American Heart Association's Construct of Cardiovascular Health: A Presidential Advisory From the American Heart Association,” Circulation 146 (2022): E18–E43, 10.1161/CIR.0000000000001078. 35766027 PMC10503546

[14] N. S. Shetty , V. Parcha , N. Patel , et al., “AHA Life's Essential 8 and Ideal Cardiovascular Health Among Young Adults,” American Journal of Preventive Cardiology 13 (2023): 100452, 10.1016/j.ajpc.2022.100452. 36636126 PMC9830108

[15] C. Li , Y. Li , M. Zhao , C. Zhang , P. Bovet , and B. Xi , “Using the New ‘Life's Essential 8’ Metrics to Evaluate Trends in Cardiovascular Health Among US Adults From 2005 to 2018: Analysis of Serial Cross‐Sectional Studies,” JMIR Public Health and Surveillance 9 (2023): e45521, 10.2196/45521. 37155232 PMC10203917

[16] A. C. S. V. Motta , K. Bousquet‐Santos , I. H. L. Motoki , and J. M. D. L. Andrade , “Prevalence of Ideal Cardiovascular Health in the Brazilian Adult Population ‐ National Health Survey 2019,” Epidemiologia e Serviços de Saúde 32 (2023): e2022669, 10.1590/s2237-96222023000300006. 37018816 PMC10069666

[17] I. Mannoh , M. Hussien , Y. Commodore‐Mensah , and E. D. Michos , “Impact of Social Determinants of Health on Cardiovascular Disease Prevention,” Current Opinion in Cardiology 36 (2021): 572–579, 10.1097/HCO.0000000000000893. 34397464

[18] C. D. Gardner , M. K. Vadiveloo , K. S. Petersen , et al., “Popular Dietary Patterns: Alignment With American Heart Association 2021 Dietary Guidance: A Scientific Statement From the American Heart Association,” Circulation 147 (2023): 1715–1730, 10.1161/CIR.0000000000001146. 37128940

[19] L. T. Cacau , A. Marcadenti , A. C. Bersch‐Ferreira , et al., “The AHA Recommendations for a Healthy Diet and Ultra‐Processed Foods: Building a New Diet Quality Index,” Frontiers in Nutrition 9 (2022): 575, 10.3389/FNUT.2022.804121/BIBTEX. PMC9036106 35479734

[20] E. J. Benjamin , S. S. Virani , C. W. Callaway , et al., “Heart Disease and Stroke statistics—2018 Update: A Report From the American Heart Association,” Circulation 137 (2018), https://doi.org/10.1161/CIR.0000000000000558. 10.1161/CIR.0000000000000558 29386200

[21] Á. N. Previdelli , S. C. Andrade , M. M. Pires , S. R. G. Ferreira , R. M. Fisberg , and D. M. Marchioni , “Índice de Qualidade da Dieta Revisado Para População Brasileira,” Revista de Saúde Pública 45 (2011): 794–798, 10.1590/S0034-89102011005000035. 21655703

[22] Brasil. Guia Alimentar Para a População Brasileira: Promovendo a Alimentação Saudável. 1st ed. Brasília: Ministério da Saúde; 2006.

[23] Institute of Medicine. Dietary Reference Intakes for Water, Potassium, Sodium, Chloride, and Sulfate. Washington, D.C.: National Academies Press; 2005, 10.17226/10925.

[24] WHO. Global Strategy on Diet, Physical Activity and Health. World Health Organization 2004.

[25] A. C. Sposito , B. Caramelli , F. A. H. Fonseca , et al., “IV Diretriz Brasileira Sobre Dislipidemias e Prevenção da Aterosclerose: Departamento de Aterosclerose da Sociedade Brasileira de Cardiologia,” Arquivos Brasileiros de Cardiologia 88 (2007): 2–19, 10.1590/S0066-782X2007000700002. 17515982

[26] P. M. Guenther , J. Reedy , and S. M. Krebs‐Smith , “Development of the Healthy Eating Index‐2005,” Journal of the American Dietetic Association 108 (2008): 1896–1901, 10.1016/j.jada.2008.08.016. 18954580

[27] L. T. Cacau , M. de Almeida Alves , I. de Souza Santos , et al., “Prospective Association Between the Cardiovascular Health Diet Index and Subclinical Atherosclerosis: The Brazilian Longitudinal Study of Adult Health (ELSA‐Brasil) Cohort Study,” British Journal of Nutrition 132 (2024): 1637–1644, 10.1017/S0007114524002836. 39523207

[28] L. T. Cacau , P. A. Lotufo , I. M. Benseñor , and D. M. Marchioni , “Dietary Patterns Derived From Three Diet Quality Indices and Associated Factors: Findings From the Baseline of ELSA‐Brasil,” Ciência & Saúde Coletiva 30 (2025): e14412023, 10.1590/1413-81232025306.14412023. 40561322

[29] T. Aloy dos Santos , V. S. Chites , B. P. Riboldi , et al., “Could the Wheel of Cardiovascular Health Diet Be a Tool for Diet Quality in Nutritional Counseling? Comparison With Healthy Eating Index‐2020,” Journal of the American Nutrition Association 43 (2024): 376–383, 10.1080/27697061.2023.2297888. 38175725

[30] R. S. de Oliveira Neta , S. C. V. C. Lima , M. F. A. Medeiros , et al., “The EAT‐Lancet Diet Associated Cardiovascular Health Parameters: Evidence From a Brazilian Study,” Nutrition Journal 23 (2024): 116, 10.1186/S12937-024-01021-4/TABLES/4. 39354466 PMC11443638

[31] A. C. Bersch‐Ferreira , R. H. V. Machado , J. S. Oliveira , et al., “A Nutritional Strategy Based on Multiple Components for Glycemic Control in Type 2 Diabetes: A Multicenter Randomized Controlled Clinical Trial,” Nutrients 16 (2024): 3849, 10.3390/NU16223849/S1. 39599635 PMC11597113

[32] R. Medronho , A. de , K. V. Bloch , R. R. Luiz , and G. L. Werneck Epidemiologia. 2nd ed. Rio de Janeiro: Atheneu; 2008.

[33] W. G. Cochran Técnicas de Amostragem. 2nd ed. Portugal: Fundo de Cultura; 1963.

[34] D. Machin , M. J. Campbell , S. B. Tan , and S. H. Tan Sample Size Tables for Clinical Studies. 3rd ed. Chichester, UK: Wiley‐Blackwell; 2009.

[35] C. S. Costa , F. R. Faria , K. T. Gabe , et al., “Escore Nova de Consumo de Alimentos Ultraprocessados: Descrição e Avaliação de Desempenho no Brasil,” Revista de Saúde Pública 55 (2021): 13, 10.11606/s1518-8787.2021055003588. 33886951 PMC8023324

[36] C. A. Monteiro , G. Cannon , R. B. Levy , et al., “Ultra‐Processed Foods: What They Are and How to Identify Them,” Public Health Nutrition 22 (2019): 936–941, 10.1017/S1368980018003762. 30744710 PMC10260459

[37] A. J. Moshfegh , D. G. Rhodes , D. J. Baer , et al., “The US Department of Agriculture Automated Multiple‐Pass Method Reduces Bias in the Collection of Energy Intakes,” American Journal of Clinical Nutrition 88 (2008): 324–332, 10.1093/ajcn/88.2.324. 18689367

[38] J. E. Cade , M. Warthon‐Medina , S. Albar , et al., “DIET@NET: Best Practice Guidelines for Dietary Assessment in Health Research,” BMC Medicine 15 (2017): 202, 10.1186/S12916-017-0962-X/TABLES/2. 29137630 PMC5686956

[39] A. B. V. Pinheiro , E. M. de A. Lacerda , E. H. Benzecry , M. C. da S. Gomes , and V. M. da Costa Tabela Para Avaliação de Consumo Alimentar em Medidas Caseiras. 5th ed. São Paulo: Atheneu; 2008.

[40] R. M. Fisberg and D. M. L. Marchioni Manual de Avaliação do Consumo Alimentar em Estudos Populacionais: A Experiência do Inquérito de Saúde em São Paulo (ISA). São Paulo: Faculdade de Saúde Pública da USP; 2012.

[41] S. F. Schakel , “Maintaining a Nutrient Database in a Changing Marketplace: Keeping Pace With Changing Food Products—A Research Perspective,” Journal of Food Composition and Analysis 14 (2001): 315–322, 10.1006/jfca.2001.0992.

[42] W. K. S. Barroso , C. I. S. Rodrigues , L. A. Bortolotto , et al., “Diretrizes Brasileiras de Hipertensão Arterial – 2020,” Arquivos Brasileiros de Cardiologia 116 (2021): 516–658, 10.36660/abc.20201238. 33909761 PMC9949730

[43] J. Gill Generalized Linear Models: A Unified Approach. Vol. 07–134. Thousand Oaks, CA: Sage: Sage University Papers Series on Quantitative Applications in the Social Sciences; 2000.

[44] G. Zou , “A Modified Poisson Regression Approach to Prospective Studies With Binary Data,” American Journal of Epidemiology 159 (2004): 702–706, 10.1093/AJE/KWH090. 15033648

[45] A. V. Mello , J. L. Pereira , A. C. B. Leme , M. Goldbaum , C. L. G. Cesar , and R. M. Fisberg , “Social Determinants, Lifestyle and Diet Quality: A Population‐Based Study From the 2015 Health Survey of São Paulo, Brazil,” Public Health Nutrition 23 (2020): 1766–1777, 10.1017/S1368980019003483. 31847928 PMC10200527

[46] M. Brauer , G. A. Roth , A. Y. Aravkin , et al., “Global Burden and Strength of Evidence for 88 Risk Factors in 204 Countries and 811 Subnational Locations, 1990–2021: A Systematic Analysis for the Global Burden of Disease Study 2021,” Lancet 403 (2024): 2162–2203, 10.1016/S0140-6736(24)00933-4. 38762324 PMC11120204

[47] G. Z. Longo , K. D. Ordaz , D. C. G. da Silva , et al., “Dietary Patterns and Cardiovascular Risk Factors Among Brazilians: A Population‐Based Study in Viçosa, Minas Gerais,” Nutrition 98 (2022): 111626, 10.1016/j.nut.2022.111626. 35397386

[48] T. W. Ushula , A. Mamun , D. Darssan , et al., “Dietary Patterns Explaining Variations in Blood Biomarkers in Young Adults Are Associated With the 30‐Year Predicted Cardiovascular Disease Risks in Midlife: A Follow‐Up Study,” Nutrition, Metabolism, and Cardiovascular Diseases 33 (2023): 1007–1018, 10.1016/j.numecd.2023.02.019. 36958973

[49] G. Pagliai , M. Dinu , M. P. Madarena , M. Bonaccio , L. Iacoviello , and F. Sofi , “Consumption of Ultra‐Processed Foods and Health Status: A Systematic Review and Meta‐Analysis,” British Journal of Nutrition 125 (2021): 308–318, 10.1017/S0007114520002688. 32792031 PMC7844609

[50] E. A. F. Nilson , G. Ferrari , M. L. C. Louzada , R. B. Levy , C. A. Monteiro , and L. F. M. Rezende , “The Estimated Burden of Ultra‐Processed Foods on Cardiovascular Disease Outcomes in Brazil: A Modeling Study,” Frontiers in Nutrition 9 (2022): 1043620, 10.3389/FNUT.2022.1043620/FULL. 36466395 PMC9712187

[51] G. Bennett , L. A. Bardon , and E. R. Gibney , “A Comparison of Dietary Patterns and Factors Influencing Food Choice Among Ethnic Groups Living in One Locality: A Systematic Review,” Nutrients 14 (2022): 941, 10.3390/NU14050941/S1. 35267916 PMC8912306

[52] T. Agurs‐Collins , J. Alvidrez , S. ElShourbagy Ferreira , et al., “Perspective: Nutrition Health Disparities Framework: A Model to Advance Health Equity,” Advances in Nutrition 15 (2024): 100194, 10.1016/J.ADVNUT.2024.100194. 38616067 PMC11031378

[53] Z. Xu , L. M. Steffen , E. Selvin , and C. M. Rebholz , “Diet Quality, Change in Diet Quality and Risk of Incident CVD and Diabetes,” Public Health Nutrition 23 (2020): 329–338, 10.1017/S136898001900212X. 31511110 PMC6992481

[54] N. M. Isiozor , S. K. Kunutsor , A. Voutilainen , and J. A. Laukkanen , “Life's Essential 8 and the Risk of Cardiovascular Disease Death and All‐Cause Mortality in Finnish Men,” European Journal of Preventive Cardiology 30 (2023): 658–667, 10.1093/EURJPC/ZWAD040. 36753230

[55] X. Wang , Y. Bai , X. Zou , X. He , X. Xie , and J. Ru , “Exploring the Role of Life Essential 8 in Patients Diagnosed With Systemic Lupus Erythematosus and Rheumatoid Arthritis,” Clinical Rheumatology 43 (2024): 3105–3116, 10.1007/S10067-024-07114-Z/TABLES/7. 39167326 PMC11442584

[56] E. V. Junior , D. C. R. S. de Oliveira , and R. Sichieri , “Cost of Healthy and Culturally Acceptable Diets in Brazil in 2009 and 2018,” Revista de Saude Publica 55 (2021), 10.11606/S1518-8787.2021055003329. PMC8627151 34852164

[57] J. Calu Costa , A. Cristina , S. De , et al., “Differences in Food Consumption of the Brazilian Population by Race/Skin Color in 2017–2018,” Revista de Saude Publica 57 (2023): 4, 10.11606/S1518-8787.2023057004000. 36820683 PMC9933641

