# Supplementary material for: Associations Between the Cardiovascular Health Diet Index (CHDI) and Cardiometabolic Risk Factors in Brazilian Workers: A Cross‐Sectional Study
Source: J Hum Nutr Diet. 2026 Jan 20;39(1):e70206. doi: 10.1111/jhn.70206 (PMC12819359; doi:10.1111/jhn.70206)
Supplement: Supplementary file 1 — Figure S1: Components and scoring criteria of the Cardiovascular Health Diet Index (CHDI) and the Brazilian Healthy Eating Index‐Revised (BHEI‐R). Figure S2: Radar plot of dietary index component scores. Table S1: Comparison analyses between sociodemographic/clinical variables and either Cardiovascular Health Diet Index (CHDI) or Brazilian Healthy Eating Index‐Revised (BHEI‐R) scores. Campinas, June 2022 to February 2023. [file JHN-39-0-s001.docx]

**Supplemental Material**

**Table S1 –** Comparison analyses between sociodemographic/clinical variables and either Cardiovascular Health Diet Index (CHDI) or Brazilian Healthy Eating Index-Revised (BHEI-R) scores. Campinas, June 2022 to February 2023.

|  |  | **CHDI** | | | **BHEI-R** | | |
| --- | --- | --- | --- | --- | --- | --- | --- |
| **Sociodemographic characteristics** | **n** | **Median** | **IIQ** | **p *** | **Median** | **IIQ** | **p *** |
| Race |  |  |  |  |  |  |  |
| *White* | 421 | 50.3 | 23.1 | **0.0052** | 62.9 | 18.0 | **0.0183** |
| *Other* | 135 | 44.7 | 26.2 |  | 59.7 | 15.6 |  |
| **Clinical characteristics** |  |  |  |  |  |  |  |
| Hypertension |  |  |  |  |  |  |  |
| *Without* | 415 | 50.8 | 23.8 | **0.0035** | 62.3 | 17.9 | **0.0171** |
| *With* | 122 | 44.1 | 22.8 |  | 60.2 | 18.2 |  |
| Diabetes Mellitus |  |  |  |  |  |  |  |
| *Without* | 491 | 49.7 | 23.9 | **0.0405** | 61.8 | 18.0 | 0.436 |
| *With* | 34 | 43.3 | 25.0 |  | 60.0 | 17.4 |  |
| BMI |  |  |  |  |  |  |  |
| *≤ 25 Kg/m²* | 224 | 52.6 | 24.9 | **0.0038** | 62.7 | 19.2 | 0.096 |
| *> 25 Kg/m²* | 333 | 46.8 | 22.0 |  | 61.7 | 17.1 |  |
| Waist circumference |  |  |  |  |  |  |  |
| *Normal* | 217 | 52.1 | 24.5 | **0.0352** | 61.5 | 19.3 | 0.991 |
| *Higher risk* | 340 | 47.0 | 22.5 |  | 62.2 | 16.6 |  |
| Triglycerides | 456 | 50.0 | 24.3 | **0.0108** | 62.3 | 18.4 | 0.166 |
| *< 150 mg/dl* | 101 | 44.6 | 19.6 |  | 60.1 | 16.4 |  |
| *≥ 150 mg/dl* |  |  |  |  |  |  |  |
| HOMA2-IR | 207 | 54.1 | 24.2 | <**0.0001** | 62.7 | 19.5 | 0.156 |
| *≤ 1,8* | 350 | 46.1 | 23.1 |  | 61.5 | 16.4 |  |
| *> 1,8* |  |  |  |  |  |  |  |
| CRP |  |  |  |  |  |  |  |
| *Low/average* | 408 | 50.8 | 24.6 | **0.0276** | 61.6 | 18.8 | 0.581 |
| *High* | 149 | 45.9 | 19.6 |  | 62.7 | 15.6 |  |

Abbreviations: CHDI, Cardiovascular Health Diet Index; BHEI-R, Brazilian Healthy Eating Index-Revised; IQR, Interquartile range; BMI, body mass index; HOMA2-IR, Homeostatic Model Assessment for Insulin Resistance; CRP, C-reactive protein.

* p-value obtained using the Mann–Whitney test. Bold text means p < 0.05

**Figure S1 – Components and scoring criteria of the Cardiovascular Health Diet Index (CHDI) and the Brazilian Healthy Eating Index-Revised (BHEI-R).**


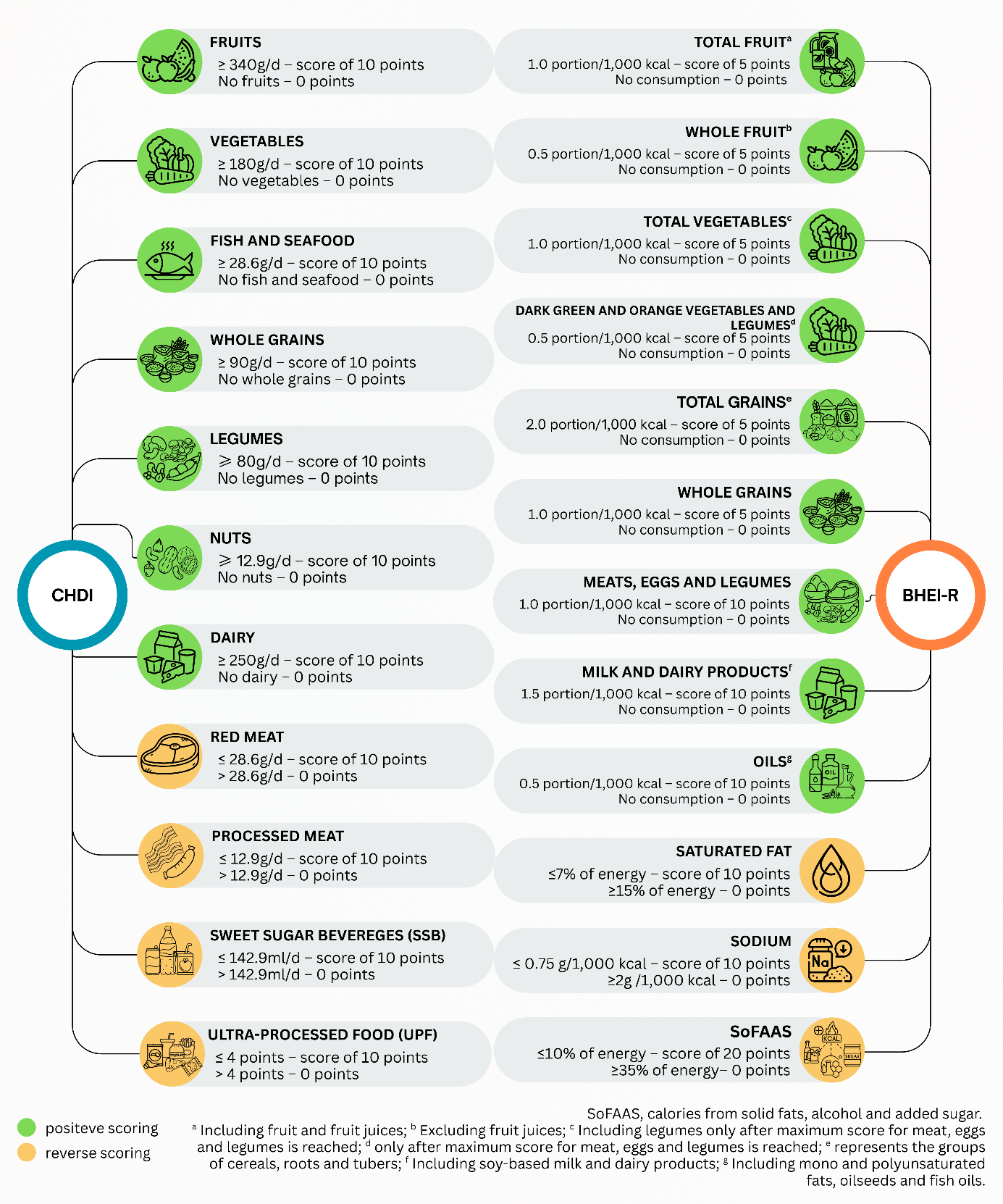


The figure illustrates the food groups, cut-off points, and maximum scores used in each index (Cardiovascular Health Diet Index (CHDI) and the Brazilian Healthy Eating Index-Revised (BHEI-R)), highlighting similarities and differences in their structure. Source: Authors’ elaboration.

**Figure S2 –** Radar plot of dietary index component scores.

**
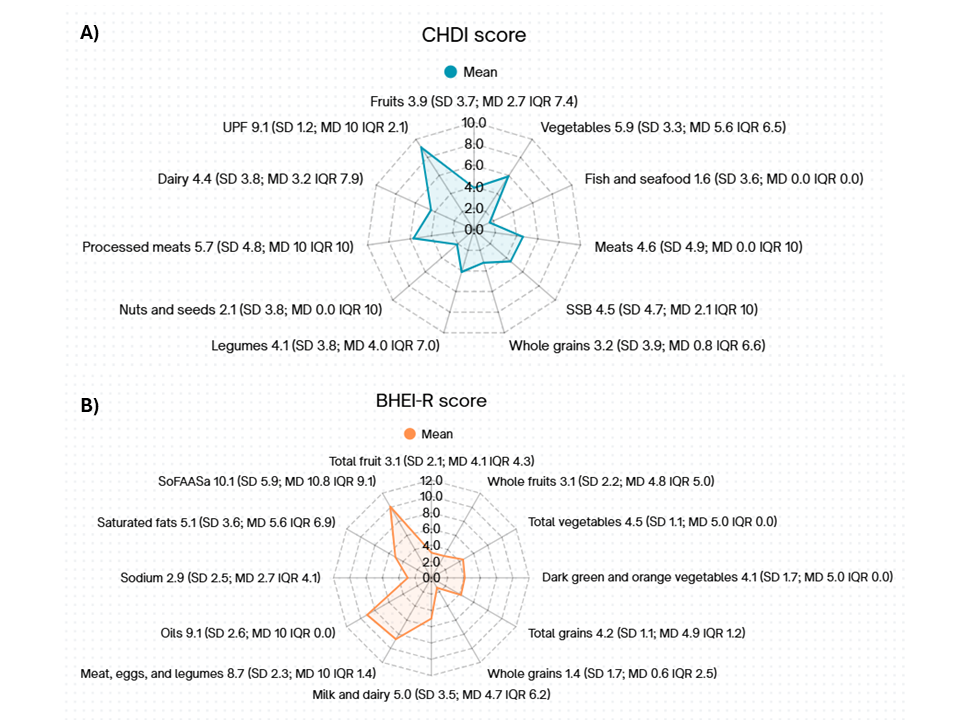
**

Radar plot showing the means, standard deviations (SD), medians (MD) and interquartile range (IQR) of the component scores for (A) the Cardiovascular Health Diet Index (CHDI) and (B) the Brazilian Healthy Eating Index-Revised (BHEI-R) among workers from a Brazilian public university (n = 557). Source: Authors’ elaboration. UPF, Ultra-processed foods; SSB, Sugar-sweetened beverages; SoFAAS, calories from solid fats, alcohol, and added sugar.
